# Enhanced Pro-Inflammatory Cytokine Responses following Toll-Like-Receptor Ligation in *Schistosoma haematobium*-Infected Schoolchildren from Rural Gabon

**DOI:** 10.1371/journal.pone.0024393

**Published:** 2011-09-08

**Authors:** Lynn Meurs, Lucja Labuda, Abena Serwaa Amoah, Moustapha Mbow, Ulysse Ateba Ngoa, Daniel Adjei Boakye, Souleymane Mboup, Tandakha Ndiaye Dièye, Adrian P. Mountford, Joseph D. Turner, Peter Gottfried Kremsner, Katja Polman, Maria Yazdanbakhsh, Ayola Akim Adegnika

**Affiliations:** 1 Department of Biomedical Sciences, Institute of Tropical Medicine, Antwerp, Belgium; 2 Department of Parasitology, Leiden University Medical Center, Leiden, The Netherlands; 3 Medical Research Unit, Albert Schweitzer Hospital, Lambaréné, Gabon; 4 Institute of Tropical Medicine, University of Tübingen, Tübingen, Germany; 5 Department of Parasitology, Noguchi Memorial Institute for Medical Research, Accra, Ghana; 6 Laboratory of Immunology, Centre Hospitalier Universitaire Le Dantec, Dakar, Senegal; 7 Department of Biology, University of York, York, United Kingdom; Ludwig-Maximilians-Universität München, Germany

## Abstract

**Background:**

*Schistosoma* infection is thought to lead to down-regulation of the host's immune response. This has been shown for adaptive immune responses, but the effect on innate immunity, that initiates and shapes the adaptive response, has not been extensively studied. In a first study to characterize these responses, we investigated the effect of *Schistosoma haematobium* infection on cytokine responses of Gabonese schoolchildren to a number of Toll-like receptor (TLR) ligands.

**Methodology:**

Peripheral blood mononuclear cells (PBMCs) were collected from *S. haematobium*-infected and uninfected schoolchildren from the rural area of Zilé in Gabon. PBMCs were incubated for 24 h and 72 h with various TLR ligands, as well as schistosomal egg antigen (SEA) and adult worm antigen (AWA). Pro-inflammatory TNF-α and anti-inflammatory/regulatory IL-10 cytokine concentrations were determined in culture supernatants.

**Principal Findings:**

Infected children produced higher adaptive IL-10 responses than uninfected children against schistosomal antigens (72 h incubation). On the other hand, infected children had higher TNF-α responses than uninfected children and significantly higher TNF-α to IL-10 ratios in response to FSL-1 and Pam3, ligands of TLR2/6 and TLR2/1 respectively. A similar trend was observed for the TLR4 ligand LPS while Poly(I:C) (Mda5/TLR3 ligand) did not induce substantial cytokine responses (24 h incubation).

**Conclusions:**

This pilot study shows that *Schistosoma*-infected children develop a more pro-inflammatory TLR2-mediated response in the face of a more anti-inflammatory adaptive immune response. This suggests that *S. haematobium* infection does not suppress the host's innate immune system in the context of single TLR ligation.

## Introduction

Schistosomiasis is a parasitic disease of major public health importance and is largely chronic in nature. The WHO estimates that more than 207 million people are infected worldwide [1], [2]. Chronic helminth infections are generally assumed to cause down-regulation of immune responses allowing long-term survival of the parasite on the one hand and minimizing immune immunopathology on the other [3] with important consequences to the host’s health [4]–[7]. Effects of schistosomal infection on adaptive immunity are widely studied in this context and it has been shown that chronic schistosomiasis inhibits *in vitro* proliferation of human lymphocytes in response to schistosomal antigens [8], [9]. The anti-inflammatory cytokine interleukin (IL)-10 plays an important role in this down-regulation [10]–[14]. Van den Biggelaar and colleagues have demonstrated that children chronically infected with schistosomes produce more IL-10 together with IL-5 and IL-13 in response to adult worm antigen (AWA), than those free of *Schistosoma* infection [15]. Helminth-induced immunomodulation may also have an impact on other infections and vaccine effectiveness. For example, in filarial infections, IL-10 down-regulates responses to unrelated antigens such as tetanus toxoid [16], [17]. Similar mechanisms might explain the impaired reaction to tetanus toxoid observed in *Schistosoma mansoni*-infected individuals [18]. Another feature of IL-10 is that it might suppress atopy in *Schistosoma haematobium*-infected children [15], [19].

Although it is becoming clearer how chronic schistosomiasis impacts on adaptive immune responses in terms of immunoregulation, the effects of schistosomal infection on innate immunity is a less studied area. In general, the innate immune system detects invading pathogens through a set of ‘pattern-recognition receptors’ (PRRs) which recognize ‘pathogen-associated molecular patterns’ (PAMPs) [20], [21]. Engagement of PRRs, such as Toll-like receptors (TLRs) initiates early immune events prior to the full activation of adaptive immunity. TLRs act as homo- (e.g. TLR3 or TLR4) or heterodimers (e.g. TLR2/1 or TLR2/6), or in combination with other PRRs such as C-type lectins. Most TLRs induce IL-12p70 which in turn promotes differentiation of Th1 cells and inflammatory responses, characterized by production of tumor necrosis factor α (TNF-α) and interferon γ (IFN-γ). On the other hand, C-type lectins can be in part responsible for the induction of anti-inflammatory responses [22]. The interplay between innate receptors is thought to form the signals that will determine the development of adaptive immune responses [21], [23].

The present study is part of the SCHISTOINIR project that aims to explore innate immune responses in schistosomiasis (for further information: www.york.ac.uk/res/schistoinir). This pilot study was set out to investigate how chronic schistosomiasis affects the peripheral blood mononuclear cell (PBMC) response to various TLR ligands. Here, we report the results from children living in a *S. haematobium*-endemic rural area near Lambaréné, Gabon.

## Methods

### Ethics statement

The study was approved by the “Comité d’Ethique Regional Independent de Lambaréné” (CERIL). Written informed consent was obtained from parents or legal guardians of all children participating prior to inclusion into the study.

### Study site and population

This pilot study was performed in Zilé, a rural area situated 16 km from Lambaréné, Gabon [24], [25]. This area is endemic for *S. haematobium*, but with important focal differences. Within Zilé there are two smaller communities, one with high (PK15) and one with low transmission (PK17), 2 km apart from each other. In June and July 2008, we recruited 17 *S. haematobium*-infected schoolchildren from PK15 and 13 uninfected from PK17, and compared their immune profiles. Study participants had no history of anti-helminthic treatment within the last 3 months prior to this study.

### Parasitological examination

*S. haematobium* infection was determined a maximum of seven days prior to blood collection by examining the residue of 10 ml of urine passed through a filter of 12-µm pore-size (Millipore). Children were classified as *S. haematobium*-infected if at least one *S. haematobium* egg was detected in the urine, or uninfected if three consecutive urine samples were negative. Geometric mean infection intensity was calculated for infected children using the first urine reading. Infections with intestinal helminths *Ascaris lumbricoides* and *Trichuris trichiura* were determined by analyzing one fresh stool sample using the Kato-Katz method 30–45 minutes after preparation [26]. *Necator americanus* larvae were detected in a 7-day coproculture of the same stool sample. To this end the classical charcoal culture procedure was used [27]–[29]. Infection with *Plasmodium falciparum* and microfilaria was determined by Giemsa-stained thick blood smears [30].

After collection of blood samples, all *S. haematobium*-infected children were treated with a single dose of praziquantel (40 mg/kg), and those with intestinal helminths were given a single dose of albendazole (400 mg). Children with other infections or clinical complaints were provided with appropriate medical care.

### Hematology

Hematological parameters were analyzed using the ADVIA® 120 Hematology System (Bayer Health Care) and erythrocyte sedimentation rate was determined manually.

### PBMC cultures

PBMCs were isolated from heparinized venous blood by density centrifugation on Ficoll (AZL pharmacy, The Netherlands) and cultured in 200 µl of IMDM supplemented with 5% fetal bovine serum (Greiner Bio-One), 100 U/ml penicillin (Astellas), 10 µg/ml streptomycin, 1 mM pyruvate and 2 mM L-glutamine (Sigma) in 96-well round bottom plates (Nunc). PBMCs were seeded at 1×10^6^ cells/well for stimulation with schistosomal egg antigen (SEA) or adult worm antigen (AWA), and at 5×10^5^ cells/well for stimulation with specific TLR ligands and phyto-hemagglutinin (PHA; positive control). IMDM medium was used as a negative control. Supernatants were collected after 24 h and 72 h of incubation at 37°C in a 5% CO_2_ atmosphere and stored at −80°C until cytokine analysis.

### Innate and adaptive stimuli

A panel of four TLR ligands was used:

1. TLR2/1 ligand, Pam3CSK4 (Pam3), a synthetic bacterial triacylated lipopeptide (final concentration of 100 ng/ml; EMC microcollections GmbH),
2. TLR2/6 ligand, FSL-1, a synthetic diacylated lipoprotein mimicking the N-terminal part of LP44 from *Mycoplasma salivarium* (50 ng/ml; InvivoGen),
3. TLR3 ligand polyinosinic-polycytidylic acid (poly(I:C)), a synthetic analogue of viral double-stranded RNA (50 µg/ml; InvivoGen),
4. TLR4 ligand, ultrapure lipopolysaccharide (LPS) derived from *Escherichia coli* (100 ng/ml; InvivoGen).

In addition to the classical innate ligands, we used the schistosomal antigen-containing products SEA and AWA from *S. haematobium* at a final concentration of 10 µg protein/ml. Purified PHA (Remel) was used as a positive control at a final concentration of 2 µg/ml.

### Cytokine analysis

Two cytokines were measured in this pilot study; one key pro-inflammatory cytokine, TNF-α [31]–[33], and the anti-inflammatory/regulatory cytokine IL-10 [12], [34]–[37]. Cytokine concentrations were measured in supernatants from single PBMC cultures by ELISA according to the manufacturer's instructions, using half of the reaction volume (PeliKine Compact^TM^, Human TNF-α and IL-10 ELISA kit, Sanquin).

A pro-inflammatory index was computed as the TNF-α: IL-10 ratio upon stimulation relative to the spontaneously produced TNF-α: IL-10 ratio (i.e. in medium):
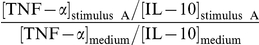



### Statistical analysis

Differences between schistosome-infected and uninfected groups were determined by the Fisher’s exact test for sex and intestinal helminth infections and by the Mann-Whitney U test for helminth infection intensity, body mass index (BMI) and hematological parameters. Age was normally distributed and differences between infection groups were tested using the independent student’s T test. As cytokine concentrations were not normally distributed, nonparametric tests were used. Differences in an individual’s cytokine response to different stimuli (e.g. medium *versus* stimulus-induced cytokine concentration, or ratio) were tested by the Wilcoxon matched-pair signed-rank test.

Differences between infection groups in cytokine concentrations and pro-inflammatory indices were tested by the Mann-Whitney U test. Cytokine concentrations were corrected for spontaneous cytokine production. This was done by subtracting the spontaneously induced cytokine concentration (i.e. in medium) from the stimulus-induced cytokine concentration. For the pro-inflammatory index, this was done by dividing the stimulus-induced ratio by the spontaneously induced ratio (see formula above). Similarly, Spearman’s ρ was calculated to estimate the correlation between cytokine responses and infection intensity.

SPSS 18.0 (SPSS Inc.) and GraphPad Prism 5 (GraphPad Software, Inc.) were used for statistical analysis. Results were considered significant when the *p*-value was <0.05.

## Results

### Characteristics of the study population

Table 1 shows that the demographic, parasitological and hematological characteristics of the infected and uninfected groups of schoolchildren were comparable. Although *S. haematobium*-infected children tended to be more anemic and to have higher eosinophil levels, there were no significant differences between the groups for any of the parameters. In addition, the prevalence and infection intensity of *A. lumbricoides*, *T. trichiura* or *N. americanus* did not significantly differ between *S. haematobium*-positive and -negative children (data not shown for infection intensity). *S. haematobium* infection intensities were low (<50 epg/10 ml) in 14/17 infected children [38]. Malaria and filariasis are endemic in this area [39]. However, only one study participant was infected with *P. falciparum* and one with microfilariae.

**Table 1:** General characteristics of the study population.

	Uninfected	Infected
N	13	17
Age (mean (range))	10.2 y (7–16)	11.4 y (7–16)
Sex (boys/girls)	8/5	9/8
BMI (median (interquartile range))	15.5 kg/m^2^ (2.83)	15.7 kg/m^2^ (1.64)
*S. haematobium* infection intensity (geometric mean (range))	Not applicable	9.8 eggs/10 ml (1–1002)
Intestinal helminth infection (n/total)	5/12	5/11
*N. americanus* infection(n/total)	1*/12	2/12
*A. lumbricoides* infection (n/total)	2**/12	2**/11
*T. trichiura* infection (n/total)	4***/12	2**/11
Malaria (n/total)	1/12	0/17
Microfilariasis (n/total)	0/12	1/17
Erythrocyte sedimentation rate (median (range))	15 mm/h (6–50)	15 mm/h (10–88)
Level of white blood cells (median (range))	8.70•10^3^/µl (3.80–10.30)	7.20•10^3^/µl (5.00–12.40)
Level of lymphocytes (median (range))	3.22•10^3^/µl (1.68–5.00)	3.33•10^3^/µl (1.26–4.60)
Level of monocytes (median (range))	0.68•10^3^/µl (0.36–1.00)	0.64•10^3^/µl (0.49–1.04)
Level of neutrophils (median (range))	2.99•10^3^/µl (1.06–6.08)	2.14•10^3^/µl (1.05–5.79)
Level of eosinophils (median (range))	0.79•10^3^/µl (0.20–1.93)	1.53•10^3^/µl (0.50–2.49)
Level of basophils (median (range))	0.09•10^3^/µl (0.04–0.27)	0.07•10^3^/µl (0.04–0.12)
Level of hemoglobin (median (range))	12.2 g/dl (11.4–13.2)	11.3 g/dl (9.1–13.6)

Thick smears (n = 1) for the diagnosis of blood parasites as well as stool samples (n = 6 for Kato-Katz and n = 5 for coproculture) for the diagnosis of intestinal helminths were missing at random.

* Including one child with a *T. trichiura* - *N. americanus* co-infection.

** Including one child with a *T. trichiura* - *A. lumbricoides* co-infection.

*** Including one child with a *T. trichiura* - *N. americanus* co-infection and another one with a *T. trichiura* - *A. lumbricoides* co-infection.

### Cytokine responses to TLR-ligands

**Figure 1:**
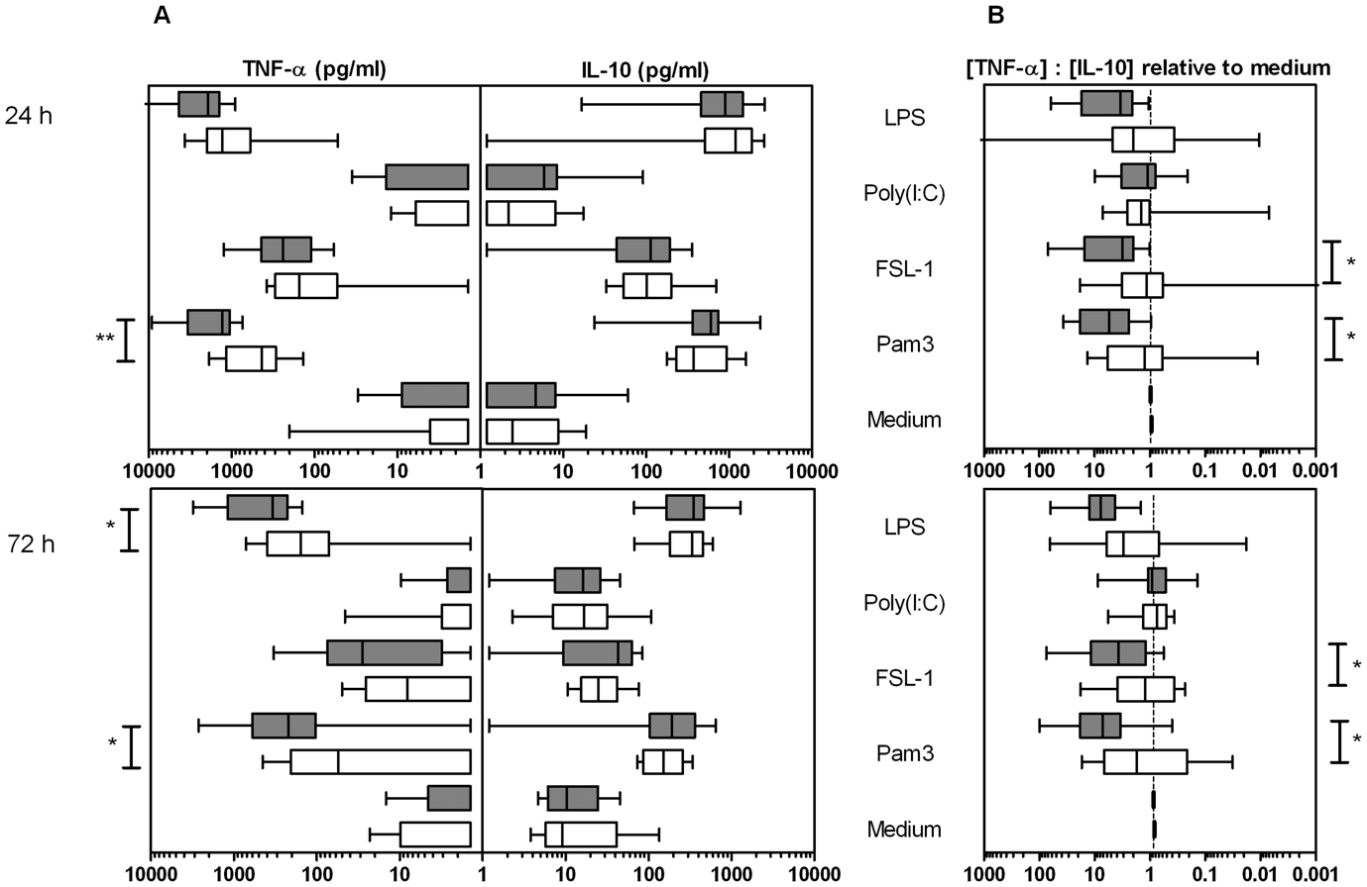
Cytokine production in response to TLR ligands in PBMC cultures. White and grey boxes correspond to *S. haematobium*-free and infected children respectively with the whiskers indicating minimal and maximal concentrations. * *p*<0.05; ** *p*<0.01. **Panel A**: At 24 h, the ‘innate’ time-point, infected children produced significantly more TNF-α in response to the TLR2/1 ligand Pam3CSK4 (Pam3) compared with uninfected children. TLR-mediated IL-10 production did not significantly deviate between infection groups. When innate cytokine responses faded at 72 h, similar trends were observed. These plots were not adjusted for spontaneous cytokine production. **Panel B**: At both time points, pro-inflammatory indices (i.e. cytokine ratio induced by one of the stimuli relative to the spontaneously produced ratio) induced by the TLR2 ligands, Pam3 and FSL-1, were significantly higher in infected *versus* uninfected children.

TLR-induced cytokine production by PBMC cultures from infected and uninfected groups was measured at 24 h (Figure 1A, upper panel). While Pam3, FSL-1 and LPS led to substantial and significant cytokine production in both groups compared to medium control, the TLR3 ligand poly(I:C) did not stimulate significant levels of TNF-α or IL-10. Pam3, FSL-1 and LPS tended to induce the production of greater quantities of the pro-inflammatory cytokine TNF-α in infected compared to uninfected children (Pam3; *p*<0.01), while the production of IL-10 was comparable between the two groups.

There was a stronger pro-inflammatory index for PBMC responses of infected children (Figure 1B, upper panel) upon TLR-stimulation (except poly(I:C)) than those from uninfected children. These differences were significant for both Pam3 and FSL-1 (*p*<0.05) which are ligands for TLR2.

Furthermore, *S. haematobium* infection intensity was positively associated with TLR-2- and TLR-4-mediated TNF-α but not with IL-10 responses. This was significant for Pam3 (ρ = 0.80) and FSL-1 (ρ = 0.66). Also, pro-inflammatory indices tended to be positively associated with infection intensity (significant for Pam3 with ρ = 0.60).

In order to get an idea of the dynamics of the innate response, TLR responses were also measured after 72 h. Cytokine responses to TLR ligands decreased over time, being less pronounced after 72 h. Nevertheless, the difference between infected and uninfected subjects was similar to that observed in 24 h cultures with a largely pro-inflammatory TLR response in infected subjects (Figure 1, lower panels).

### Cytokine responses to schistosomal products

**Figure 2:**
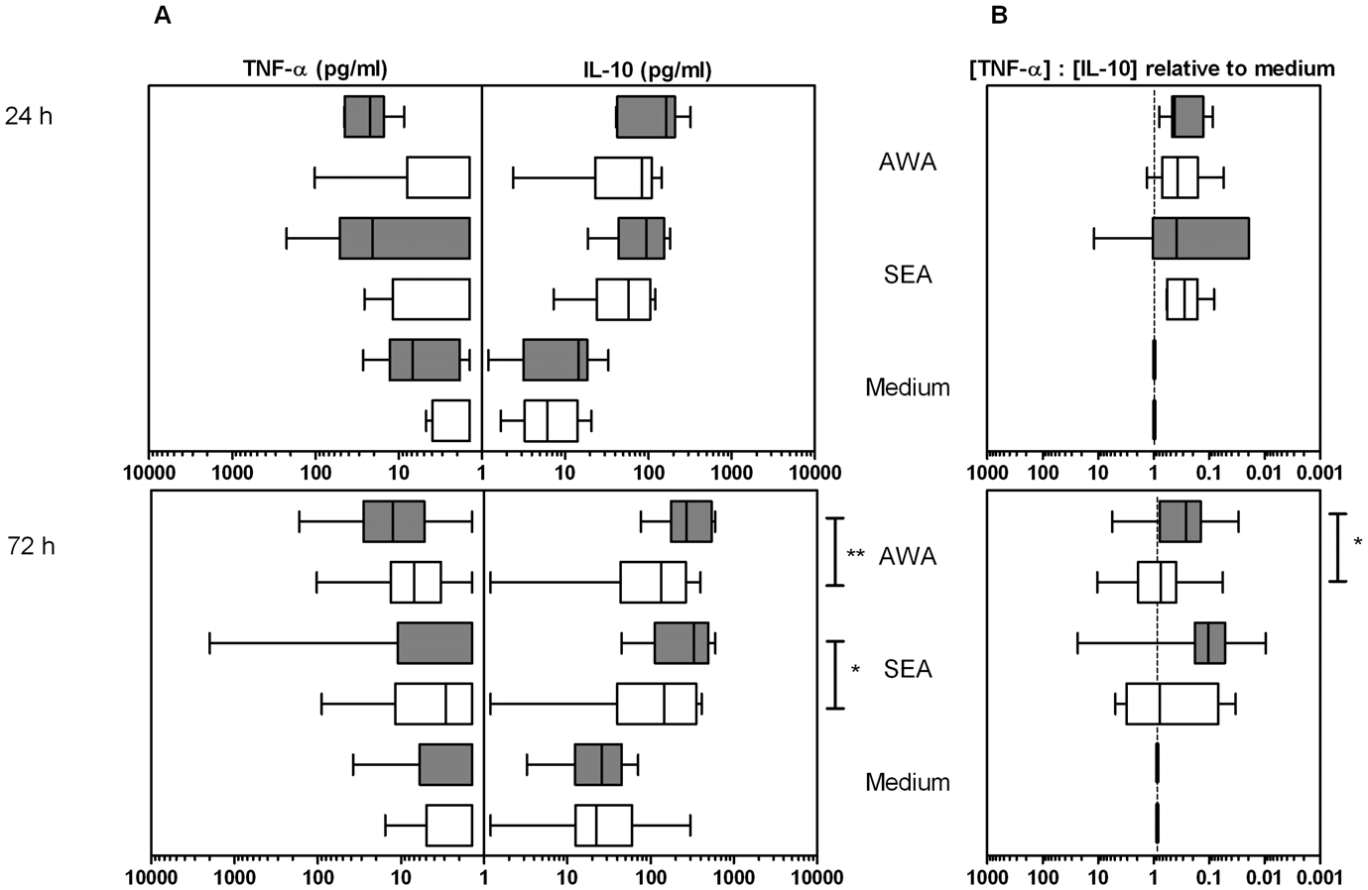
Cytokine production in response to schistosomal products in PBMC cultures. PBMCs were stimulated with schistosomal egg antigen (SEA) or adult worm antigen (AWA). White and grey boxes correspond to *S. haematobium*-free and infected children respectively with the whiskers indicating minimal and maximal concentrations. * *p*<0.05; ** *p*<0.01. **Panel A**: Cytokine production did not differ between groups at 24 h (mainly innate response) but the adaptive IL-10 response after 72 h of incubation to both schistosomal products was significantly higher in infected children than in *S. haematobium*-free children. Furthermore, only infected children produced a significant innate TNF-α response to schistosomal products at 24 h. These plots are not adjusted for spontaneous cytokine production. **Panel B**: After 24 h (predominantly innate), most cytokine ratios induced by schistosomal products were more anti-inflammatory than the ratios induced by medium alone. When the adaptive response had developed after 72 h however, only infected children produced significant anti-inflammatory cytokine balances while pro-inflammatory indices were lower in infected children than in uninfected children (AWA; *p*<0.05).

We examined adaptive immune responses by analyzing cytokine production by PBMCs after 72 h of incubation with schistosomal antigens (Figure 2, lower panels). Since these products not only contain antigenic components but also innate ligands [40]–[47], cytokine production was assessed at both 24 h (innate) and at 72 h (adaptive response).

SEA- and AWA-induced IL-10 was detected in PBMC culture supernatants after 24 h and increased further in 72 h cultures. IL-10 production in infected children was consistently higher than in uninfected children at 24 h but this difference was only significant at 72 h: both SEA- and AWA-induced IL-10 responses were significantly higher in infected children at 72 h (Figure 2A).

After 24 h, AWA-stimulated cultures from infected children secreted significant levels of TNF-α (*p*<0.05 *cf.* medium), while in uninfected children, antigen-induced TNF-α was only detected after 72 h.

After 24 h, the cytokine ratios following stimulation with schistosomal products seemed more anti-inflammatory than pro-inflammatory (i.e. increased IL-10: TNF-α; Figure 2B, upper panel). After 72 h, the pro-inflammatory index tended to increase in uninfected children, and was significantly higher than in infected children upon stimulation with AWA (Figure 2B, lower panel).

## Discussion

In this pilot study we investigated the effects of chronic *Schistosoma* infection on TLR-mediated cytokine production. We showed that innate TNF-α responses and TNF-α: IL-10 ratios upon TLR2 stimulation of PBMCs were significantly higher in *S. haematobium*-infected children compared with those without infection, in the face of enhanced regulatory adaptive responses to schistosomal antigens. This suggests that schistosomal infection is associated with elevated pro-inflammatory TLR2 responses.

While cytokine responses upon TLR stimulation tended to decrease over time, the reverse was seen after stimulation with schistosomal products. Although at 24 h, TNF-α levels tended to be more pronounced in infected than in uninfected children, analogous with the observed TLR-mediated cytokine responses, after 72 h of stimulation, PMBCs from *S. haematobium*-infected children produced significantly more IL-10 than uninfected children, as has been described elsewhere [15]. Schistosomal products are more complex than single TLR stimuli. SEA and AWA stimulate the innate immune system through TLRs, C-type lectins and other innate receptors [40]–[47] but in addition, they contain antigens which can be processed and presented to the T cell receptor forming the basis of acquired immune responses. Schistosomal products are thus able to activate both innate and adaptive pathways. This may explain the observed trend of an initial pro-inflammatory-like immune response. Concurrent with a fading innate cytokine response, a clear anti-inflammatory adaptive response comprising elevated IL-10 was detected in infected children at 72 h.

To our knowledge there are only two other human studies on TLR-mediated cytokine profiles in schistosomiasis. Van der Kleij *et al.* reported reduced TLR responses in Gabonese *S. haematobium*-infected as compared to uninfected children [24]. These results seem to contradict the current findings, but can be explained by differences in the selection of the uninfected control groups. While in the present study both infected and uninfected children were from the same *S. haematobium-*endemic rural area (Zilé), Van der Kleij *et al.* recruited the uninfected control group from a non-endemic neighboring semi-urban area to ensure that the negative subjects were truly negative with no history of exposure. However, when we examined the dataset of the Van der Kleij study to compare the infected (n = 5) and uninfected (n = 10) groups from the same rural area of Zilé, infected children tended to produce higher TNF-α levels as well as higher pro-inflammatory indices in response to LPS than their uninfected counterparts, which is in line with our results.

The second study of TLR-mediated cytokine profiles was carried out in Brazil and also corresponds with our results. Although they did not highlight it, Montenegro *et al.* showed that 48 h whole blood cultures from *S. mansoni*-infected adults produced more TNF-α than those of uninfected adults in response to LPS [48].

In addition, a mouse model of infection pointed towards a similar association between TLR function and schistosomal infection and suggests that schistosomal infection induces elevated pro-inflammatory TLR responses [49].

Few field studies on TLR responses in other helminth infections than schistosomiasis have been published and show varying results. In contrast to *S. haematobium* infection, a negative association has been found between infection with the filarial nematode *Wuchereria bancrofti* and intracellular pro-inflammatory cytokine expression upon TLR stimulation in lymphocytes and monocytes in a population in South India [50], [51]. In addition, *W. bancrofti* was associated with reduced pro-inflammatory cytokine responses upon TLR stimulation of PBMCs in a population from the same area with latent tuberculosis [52]. On the other hand, Jackson *et al*. showed infections with intestinal nematodes, i.e. hookworm and *Trichuris trichiura*, to be associated with pro-inflammatory cytokine production, but not with regulatory cytokine production, upon TLR2 and TLR4 stimulation of monocytes from children from Pemba Island [53]. Apparently, the effect of helminth infections on the innate immune responses is species specific; some helminth species may be associated with more pro-inflammatory TLR responses while other helminths are associated with more anti-inflammatory TLR responses. In the present study, *S. haematobium*-infected and uninfected children did not significantly differ with respect to the number and species of other helminth infections. However, information on soil-transmitted helminth infections was not available for some of the participants and we cannot entirely rule out that these infections may have altered the observed innate cytokine responses. In addition, other parasites such as malaria may influence immune balances [54]. The duration as well as the intensity of infection might further influence innate immune responses. It is therefore important to confirm our findings in a larger study population and in varying epidemiological settings.

In conclusion, this pilot study on innate immune responses in schistosomiasis shows a more pro-inflammatory response to single TLR2 ligands in the face of an anti-inflammatory adaptive immune response in *S. haematobium*-infected children. Whilst the precise biological mechanisms for these observations remain to be ascertained, it seems that the commonly accepted view that schistosomal infection suppresses the host's immune system does not hold for ligation of single TLRs. Many receptors – TLRs as well as non-TLRs such as C-type lectins – are involved in innate signaling and their interactions as well as down-stream pathways are at the basis of the immune response to invading pathogens [21], [55]. Given the fact that a *Schistosoma* worm is a complex mix of ligands stimulating the innate immune system, further research into this innate cross-talk is necessary.
